# Delivery of Lutein by Using Modified Burdock Polysaccharide Aggregates: Preparation, Characterization, and In Vitro Release Properties

**DOI:** 10.3390/polym16141982

**Published:** 2024-07-11

**Authors:** Chenchen Zhang, Yan Zhang, Jiangfeng Song, Hongjuan Wang, Caie Wu, Ying Li

**Affiliations:** 1 Institute of Agro-Product Processing, Jiangsu Academy of Agricultural Sciences, Nanjing 210014, China; 2 School of Food and Biological Engineering, Jiangsu University, Zhenjiang 212013, China; 3 College of Light Industry and Food Engineering, Nanjing Forestry University, Nanjing 210037, China; 4 College of Food Science and Technology, Nanjing Agricultural University, Nanjing 210014, China

**Keywords:** lutein, burdock polysaccharide, stearic acid, aggregates, release properties

## Abstract

Novel self-assembled aggregates of stearic acid (SA)-modified burdock polysaccharide (BP) for loading lutein were constructed, and the release and absorption properties of lutein in the aggregates in simulated gastrointestinal fluid were investigated. Three different degrees of substitution (DS) of SA-BPs were used to embed lutein, resulting in the encapsulation efficiency exceeding 90%. The aggregates were uniformly spherical, with a particle size range of 227–341 nm. XRD analysis revealed that lutein was present in a non-crystalline state within the aggregates. FT-IR and FS analysis demonstrated that lutein was located in the hydrophobic domains of SA-BP. The highest bioavailability of lutein in these aggregates reached 4.36 times that in the unmodified samples. These aggregates were able to remain stable in gastric juice and enhance the release rate of lutein in intestinal fluid. The transport of lutein-loaded SA-BP aggregates in Caco-2 cells competed with P-glycoprotein inhibitors, mainly promoting the transmembrane absorption of lutein through caveolae (or lipid raft)-related and clathrin-dependent endocytosis pathways. The above results suggest that SA-BP aggregates have the potential to be promising carriers for the efficient delivery of hydrophobic lutein.

## 1. Introduction

Lutein is a xanthophyll carotenoid that naturally occurs in dark green leafy vegetables and plays an important role in preventing age-related macular degeneration (AMD), cataracts, atherosclerosis and cardiovascular diseases. The ocular-protective, anti-oxidative and anti-inflammatory activity of lutein might be the key to its health supplement effects [1]. However, the multiple hydrophobic groups in the molecular structure of lutein make it difficult to dissolve in the aqueous phase, reducing its absorption and utilization [2]. The numerous conjugated double bonds also make it vulnerable to decomposition and inactivation, resulting in poor stability [3]. Therefore, the establishment of an effective delivery system has been considered a promising strategy to improve its potential application and bioabsorption [4].

Recently, amphiphilic polymer materials have demonstrated the ability to self-assemble into various functional micro/nanostructures for the delivery of hydrophobic bioactive substances. Amphiphilic polymer aggregates have attracted significant attention due to their affordability, biocompatibility, ease of preparation, and effectiveness [5]. For example, Chen et al. [6] synthesized amphiphilic conjugates by conjugating curcumin with hydrophilic hydroxyethyl starch, which then self-assembled to form uniform nanoparticles (HES-CUR NPs). HES-CUR NPs increased the solubility of curcumin to thousands of times that of free curcumin and effectively protected the loaded curcumin from degradation under UV light and high temperature. It also significantly improved the cell compatibility and antioxidant activity of curcumin. Yang et al. [7] developed a delivery system based on chitosan-stearic acid polymer carriers to improve the oral bioavailability of paclitaxel. The intracellular uptake of the Caco-2 cell model verified the improvement of paclitaxel intestinal absorption. Compared to free paclitaxel, the bioavailability of paclitaxel was increased by 1.66 times after the embedding of the polymer carriers. The distinctive hydrophobic domains of amphiphilic biopolymer aggregates present opportunities for encapsulating hydrophobic and sensitive bioactive components.

Among the polymeric aggregates, amphiphilic polysaccharides have emerged as a research focus in the current fields of food science and nutrition. They possess appealing characteristics, including self-assembly, special solubility, interfacial activity, drug-loading potential, and good biocompatibility [8]. Polymer aggregates self-assembled from hydrophobic polysaccharides can be used as targeted drug delivery nanosystems.

Abundant resources, non-toxicity, good biocompatibility, high biodegradability, and ease of structural modification make natural polysaccharides excellent materials for the preparation of hydrophobic modified polysaccharides [8]. The hydroxyl groups of natural water-soluble polysaccharides (such as dextran and fructan) can react with various organic acids and their derivatives (such as stearic acid, octenyl succinic acid, and dodecenyl succinate), thereby obtaining hydrophobic modified polysaccharides [9]. Because of the intermolecular and/or intramolecular hydrophobic interactions in aqueous media, amphiphilic polysaccharides tend to form self-aggregated copolymers.

Burdock polysaccharide (BP) is one of the important active ingredients in burdock (*Arctium lappa* L.) root. It is composed of the α-D-glucopyranosyl-(1→2)-[β-D-furanofructose-(1→2)]10-β-D-furan fructose group, as characterized by nuclear magnetic resonance spectroscopy [10]. BP exhibits excellent biocompatibility and biodegradability, making it a potential candidate for the application of hydrophobic active ingredients. It has been demonstrated to have antioxidant, anti-inflammatory, and anti-diabetic effects, as well as the ability to regulate intestinal microbiota [11]. However, BP’s water-soluble polymer property limits its application as a carrier for poorly water-soluble bioactives.

In this study, a stearic acid-modified BP copolymer was synthesized by covalently linking stearic acid to the polysaccharides, and lutein-loaded BP aggregates were obtained through the anti-solvent precipitation and dynamic high-pressure microfluidization (DHPM) method. The physicochemical and structural properties, in vitro release, and absorptive transport characteristics of these aggregates were investigated, with the aim of providing theoretical and technical support for the development of polysaccharide-based lutein nutritional excipients.

## 2. Materials and Methods

### 2.1. Materials and Reagents

The neutral polysaccharide (>85%) from burdock (*Arctium lappa* L.) root was self-made in the laboratory. Lutein (≥95%) was purchased from Shanghai Yuanye Biotechnology Co., Ltd. (Shanghai, China). The 3-(ethyliminomethylideneamino)-N, N-dimethylpropan-1-amine, hydrochloride (EDC), dimethylaminopyridine (DMAP) and stearic acid (SA) were acquired form Shanghai McLean Biochemical Co., Ltd. (Shanghai, China). Dimethyl sulfoxide (DMSO), anhydrous ethanol and n-hexane were obtained from Nanjing Chemical Reagent Co., Ltd. (Nanjing, China). Pepsin (from porcine gastric mucosa, ≥250 U/mg), trypsin, pancreatin (from porcine pancreas) and pig bile salt (≥65%) were obtained from China National Pharmaceutical Group Chemical Reagent Co., Ltd. (Shanghai, China). Dulbecco’s modified Eagle medium (DMEM), fetal bovine serum (FBS), phosphate buffer saline (PBS) and Hank’s balanced salt solution (HBSS) were all purchased from Thermo Fisher Scientific Co., Ltd. (Waltham, MA, USA). Double distilled water was prepared in our laboratory. All the other reagents were of analytical purity grade and commercially obtained.

### 2.2. Preparation of Lutein-Loaded SA-Modified BP Aggregates

#### 2.2.1. Preparation of BP

According to the previous method of Zhang et al. [12], dried burdock root was pulverized and then soaked in 95% ethanol, and lyophilized through a 200-mesh sieve. Next, 5 g of burdock powder was extracted in hot water, and the resulting polysaccharide extract was centrifuged at 4500 r/min for 20 min. The supernatant was collected and concentrated under reduced pressure, and then four times the volume of absolute ethanol was added. Overnight at 4 ° C, the precipitate was collected and washed with absolute ethanol and acetone two to three times, respectively. The crude BP was then deproteinized by the Sevag method, further decolorized by activated carbon, and then precipitated with absolute ethanol. The refined BP was washed with acetone and then freeze-dried.

#### 2.2.2. Synthesis of SA-Modified BP

By using EDC and DMAP as catalysts, the hydrophobic side chain of SA was grafted onto the framework of BP. Briefly, 1.0, 2.0, and 3.0 mmol of SA were accurately weighed, and a certain molar ratio of DMAP and EDC (SA: DMAP: EDC = 1:1:1.2) was added and dissolved together in 3 mL DMSO, respectively. The mixture was stirred and activated at 25 °C for 2 h. Then, the activated solution was slowly added to 20 mL of 0.15 g/mL BP DMSO solution, and the reaction was carried out at 55 °C for 2 h. After the reaction was ceased, the reaction solution was pipetted dropwise into anhydrous ethanol for precipitation. The precipitate was centrifuged at 5000 rpm for 30 min and washed with anhydrous ethanol 2–3 times. The white precipitate was collected and redissolved in double-distilled water. The resulting solution was transferred to a dialysis bag (MWCO = 8000–12,000 Da) for dialysis, and the dialyzed sample was collected and freeze-dried to obtain SA-modified BP (SA-BP). The synthetic route map is shown in Figure S1. According to the calculation by the method of Varavinit et al. [13], the substitution degrees (DS) of the three esterificated products (SA-BP1, SA-BP2, and SA-BP3) were 0.02393, 0.0488, and 0.08829, respectively.

#### 2.2.3. Preparation of Lutein-Loaded SA-BP Aggregates

SA-BP1, SA-BP2, and SA-BP3 were diluted individually to a concentration of 1 mg/mL. Next, 5 mL of lutein ethanol solution with a mass concentration of 1.0 mg/mL was slowly injected into 200 mL of SA-BP solution. Subsequently, the mixture was stirred for 30 min and then treated twice with a dynamic high-pressure microfluidizer (LM20, Microfluidics International Corporation, Westwood, MA, USA) at a pressure of 140 MPa. The ethanol was removed by rotary evaporation under reduced pressure. The lutein-loaded SA-BP aggregates (Lut-SA-BP) were obtained through centrifugation to remove the unloaded drug. The Lut-SA-BP aggregates prepared with three degrees of substitution of SA-BP were named Lut-SA-BP1, Lut-SA-BP2, and Lut-SA-BP3, respectively. Under the stated conditions, unmodified BP mainly formed a nanodispersion system with lutein (Lut-BP).

### 2.3. Lutein Encapsulation Efficiency and Loading Capacity

First, 2 mL of Lut-SA-BP aggregates solution was taken and thoroughly mixed with 1.0 mL of n-hexane. The mixture was centrifuged at 7500 r/min for 5 min. The supernatant was diluted to 10 mL, and the absorbance at 446 nm was measured using a UV spectrophotometer [14]. The free lutein content was calculated based on the standard curve. The content of total lutein was determined by the acetone solubilization method [15]. The encapsulation efficiency (EE) was calculated using Equation (1). The loading capacity (LC) of lutein was calculated according to Equation (2).

(1) $$\mathrm{EE}\;(\%)=\frac{\mathrm{Total}\mathrm{lutein}\mathrm{content}\left(\mathrm{mg}\right)-\mathrm{Unembedded}\mathrm{lutein}\mathrm{content}\;(\mathrm{mg})}{\mathrm{Total}\mathrm{lutein}\mathrm{content}\;(\mathrm{mg})}\times 100$$

(2) $$\mathrm{LC}\;(\%)=\frac{\mathrm{Total}\mathrm{lutein}\mathrm{content}\left(\mathrm{mg}\right)-\mathrm{Unembedded}\mathrm{lutein}\mathrm{content}\;(\mathrm{mg})}{\mathrm{Total}\mathrm{mass}\mathrm{of}\mathrm{aggregates}\;(\mathrm{mg})}\times 100$$

### 2.4. Structural Characterization

Particle size and zeta potential: The particle diameter of the Lut-SA-BP aggregates was assessed on a dynamic light scattering particle size analyzer (NICOMP Z3000, Particle Sizing Systems, Santa Barbara, FL, USA) with a scattering angle of 90° at 25 °C. Additionally, the zeta potential was measured using the same equipment.

Scanning electron microscopy (SEM) observation: One drop of the Lut-SA-BP aggregates solution was taken and fixed on a conductive gel. The sample was then sprayed with gold and the microstructure was observed with a scanning electron microscope (EVO-LS10, Zeiss Technology Co., Ltd., Oberkochen, Germany) under an accelerated voltage of 5 kV.

X-ray diffraction (XRD) analysis: The diffractogram of the Lut-SA-BP aggregate powders was investigated using a D2 PHASER D8-ADVANCE X-ray diffractometer (Bruker Corporation, Karlsruhe, Germany) from 5 to 50° (2ϴ) in steps of 0.02° using Cu-Kα radiation. The scanning tube voltage was 30 kV, the tube current was 100 mA, and the scanning rate was 0.05 °/min.

Differential scanning calorimetry (DSC) analysis: First, 5 mg freeze-dried powder was put into the aluminum crucible and raised from room temperature to 250 °C at the speed of 10 °C/ min in a nitrogen atmosphere. The nitrogen flow rate was 300 mL/h. Thermal analysis was carried out under these conditions by differential scanning calorimetry (DSC Q20, TA Instruments, New Castle, DE, USA).

Fourier-transform infrared spectroscopy (FT-IR) analysis: FTIR spectra of the Lut-SA-BP aggregate powders were obtained using a FTIR spectrometer (Nicolet iS50, Thermo Fisher Scientific, Waltham, MA, USA) with the KBr pellet method in the range of 500–4000 cm^−1^. A total of 32 spectral scans were performed. The resolution was set to 4 cm^−1^.

Fluorescence spectrum (FS) analysis: The fluorescence spectrum of the Lut-SA-BP aggregates was measured on a fluorescence spectrophotometer (LS55, PerkinElmer Inc., Waltham, MA, USA). The test sample solution was diluted to 1 mg/mL with deionized water, and 200 µL diluted sample solution was transferred to a 96-well black microplate. The parameter settings were as follows: λex = 446 nm, slit widths of 5 nm and 2 nm, scanning range: 450–600 nm.

### 2.5. Storage Stability

A certain amount of the freeze-dried samples was taken and dissolved in double distilled water to investigate the effects of natural light, dark avoidance, and storage temperature (4 °C, 25 °C, and 50 °C) on the retention rate of lutein in the aggregates solution during storage. Samples were taken at intervals, the lutein content in the sample was measured, and the lutein retention rate was calculated according to Equation (3).

(3) $$Lutein\;retention\;(\%)=\frac{The\;residue\;amount\;of\;lutein\;in\;the\;aggregates\;after\;storage\;(mg)}{The\;initial\;amount\;of\;lutein\;in\;the\;aggregates\;(mg)}\times 100$$

### 2.6. In Vitro Simulated Digestion Test

#### 2.6.1. In Vitro Simulation of Gastrointestinal Digestion

In vitro digestion simulation was modified slightly with the method of Mutsokoti et al. [16].

Simulation of gastric digestion: 40 mL simulated gastric juice (containing 140 mmol/L NaCl, 5 mmol/L KCl, 10 mmol/L CaCl_2_ • H_2_O and 3.5 mmol/L KH_2_PO_4_) was prepared, the pH was adjusted to 1.2 with 1.0 mol/L HCl, pepsin was added to a final concentration of 3.2 mg/mL, and it was kept in a 37 °C water bath for 5 min. Next, 40 mg of freeze-dried powders was mixed thoroughly with the gastric juice and digested in a shaking water bath at 37 °C for 3.0 h. After that, 2 mL was taken at 1.0, 2.0, and 3.0 h, respectively, and an equal volume of simulated gastric juice was added after each sample.

Simulation of intestinal digestion: The pH of the above gastric digestion solution was adjusted to 7.0 with 0.5 mol/L NaOH, 20 mL of intestinal fluid (containing 5 mg/mL trypsin, 31 mg/mL pig bile salt and 0.1 mol/L NaHCO_3_) was added, and it was digested in a shaking incubator at 37 °C for 8 h. Additionally, 2 mL was taken out every 1 h, and an equal volume of simulated intestinal fluid was added after each sample.

#### 2.6.2. Determination of Lutein Bioavailability

The measurement was conducted by the method of Luo et al. [17]. The digestive fluid after intestinal digestion in Section 2.6.1 was centrifuged at 8000 r/min (4 °C) for 20 min. After centrifugation, the sample was divided into a sediment layer at the bottom, an aggregate layer in the middle, and an oil layer at the top. The lutein in the middle micelle layer and the original digestive fluid were extracted and determined, respectively, and the bioavailability of lutein was calculated using Equation (4).

(4) $$Lutein\;bioavailability\;(\%)=\frac{C_{1}}{C_{2}}\times 100$$

where C_1_ represents the concentration of lutein in the middle micelle layer and C_2_ represents the concentration of lutein in the original digestive fluid.

#### 2.6.3. Determination of Cumulative Release Rate of Lutein

The cumulative release rate of lutein was determined with a slight modification by the previous method [18].

The digestion solution of different digestion times in Section 2.6.1 was taken and immediately heated in a 90 °C water bath for 5 min to terminate the reaction. It was centrifuged at 5000 r/min for 20 min at room temperature. The mixed phase of the middle layer was then taken and the content of free lutein was measured. The cumulative release rate of lutein was calculated according to Equation (5).

(5) $$Lutein\;cumulative\;release\;rate\;(\%)=\frac{C_{t}V_{t}}{M}\times 100$$

where C_t_ is the concentration of lutein in the released medium at time t, V_t_ is the volume of the released medium at time t, and M is the total amount of lutein in the aggregates.

#### 2.6.4. Establishment of Sustained Release Kinetics Model

The relationship between the cumulative release of lutein (Q) and time (t) was explored, and the curve was fitted using Origin 2021 software to determine the type of release kinetics to which the release of aggregates belonged. The five common release mathematical models [19] are as follows:

Zero-order dynamic equation: Q(t) = at + b (6)

First-order kinetic equation: Q(t) = a(1 − e^bt^) (7)

Higuchi diffusion equation: Q(t) = at^1/2^ + b (8)

Ritger-Peppas equation: Q(t) = a(t^b^) (9)

Weibull equation: Q(t) = 1 − ln(−a × t^b^) (10)

In the Equations (6)–(10), a and b are constants.

### 2.7. Cellular Absorption and Transport Characteristics

#### 2.7.1. Establishment of Caco-2 Cell Monolayer Model

Caco-2 cells were incubated in DMEM complete medium (containing 1% dual antibody and 10% FBS) in a 37 °C incubator containing 5% CO_2_ for 24 h. When Caco-2 cells grew to 80–90% of the bottom area of the bottle, they were passed and cells with a good exponential growth phase were selected for subsequent experiments. Caco-2 cells were inoculated at a density of 4 × 10^5^ cells/mL in Transwell plates with a pore size of 0.4 μm, and incubated at 37 °C for 21 days. The Caco-2 cells in the Transwell cell culture plate were replaced every other day. The transepithelial electrical resistance (TEER) value was measured to evaluate the integrity of the Caco-2 cell layer. When the TEER value was greater than 300 Ω cm^2^, it was determined that the compactness and integrity of the monolayer cells were good, and they could be used for subsequent experiments.

#### 2.7.2. Cytotoxicity Test

The cytotoxicity of the Lut-SA-BP aggregates was investigated by CCK-8 assay [20]. Cells were seeded onto 96-well plates at an initial density of 4 × 10^5^ cells/well, which were incubated for 24 h at 37 °C in a 5% CO_2_ condition. The medium was placed by samples of various concentrations of the Lut-SA-BP aggregates (50, 100, 150, 200, 250 and 300 μg/mL) and in pace with six parallel wells. After 48 h incubation, 10 µL of CCK-8 solution was then added to each well. The medium was removed after incubating for 4 h. The absorbance value was measured at 450 nm using a microplate reader. The cell viability was calculated with Equation (11):

(11) $$Cell\;viability\;(\%)=\frac{A_{1}-A_{0}}{A_{2}-A_{0}}\text{×}100$$

where A_1_ is the absorbance value of the experimental well, A_2_ is the absorbance value of the control well, and A_0_ is the absorbance value of the blank well.

#### 2.7.3. Determination of Apparent Permeability Coefficient (P_app_)

*P_app_* was determined with slight modifications following the method of Bittermann and Goss [21]. First, 250 µg/mL of the Lut-SA-BP aggregates solution was prepared using Hanks’ balanced salt solution (HBSS). The apical (AP) and the basolateral (BL) sides of the Transwell board were washed three times with HBSS, respectively. After washing, clean HBSS was added again for a 15 min co-bath operation, and then the waste liquid was discarded.

Forward transportation (AP→BL): 0.5 mL of sample solution was added to the AP side as the supply cell, and 1 mL of HBSS was added to the BL side as the receiving cell.

Reverse transportation (BL→AP): 1 mL of the test sample solution was added to the BL side as the supply pool, and 0.5 mL of HBSS was added to the AP side as the receiving pool.

The Transwell plate with added samples was placed in a constant temperature incubator for cultivation. First, 0.5 mL (AP→BL) and 0.25 mL (AP→BL) of transfer solution were taken from the receiving cell every half hour, and corresponding HBSS was supplemented to keep the volume of the receiving cell constant for 2 h, respectively. Transport liquids from different directions were collected and lutein was extracted and determined. Papp was calculated according to Equation (12).

(12) $$P_{app}=(\frac{\mathrm{dQ}}{\mathrm{dt}})\times (\frac{1}{AC_{0}})$$

where dQ/dt is the steady-state flux of lutein, µg/s; A is the surface area of the membrane, cm^2^; C_0_ is the initial concentration of lutein, µg/mL.

#### 2.7.4. Cell Uptake Inhibition Test

First, 50 μg/mL of verapamil, nystalin, and dynasore inhibitor solutions with serum-free culture medium were prepared. A complete monolayer membrane with a TEER value of 300 Ω cm^2^ was selected in Section 2.7.1, HBSS was added to the AP side and BL side, respectively, and equilibrated in a constant temperature incubator for 15 min. HBSS was then discarded. Next, 0.5 mL of the three different inhibitors mentioned above were added to the AP side as the supply cell, and 1 mL of serum-free culture medium was added to the BL side as the receiving cell. They were incubated for 3 h. The transport fluid was extracted from the BL side, and the Caco-2 cells on the monolayer membrane were scraped for the extraction and detection of lutein [22].

Lutein was determined according to the method of Arunkumar, Prashanth, and Baskaran [23]. Briefly, 1 mL of the sample solution was taken and thoroughly mixed with 1 mL of trichloromethane/methanol (2:1, *v*/*v*) mixture under dark conditions. Then, 2 mL of n-hexane was added and mixed well. It was centrifuged at 4000 r/min for 3 min. The supernatant was collected, the organic solvent was evaporated then dissolved in methanol, and finally detected using a microplate reader.

### 2.8. Statistical Analysis

All experiments were repeated three times, and the results were expressed as mean ± SD. Data processing was performed using Excel 2010, OriginPro 2021, and SPSS 25 software. Duncan’s test was used for analysis of variance, and *p* < 0.05 indicated significant differences.

## 3. Results and Discussion

### 3.1. Effect of SA-BP Carrier on the EE and LC of Lutein

**Figure 1:**
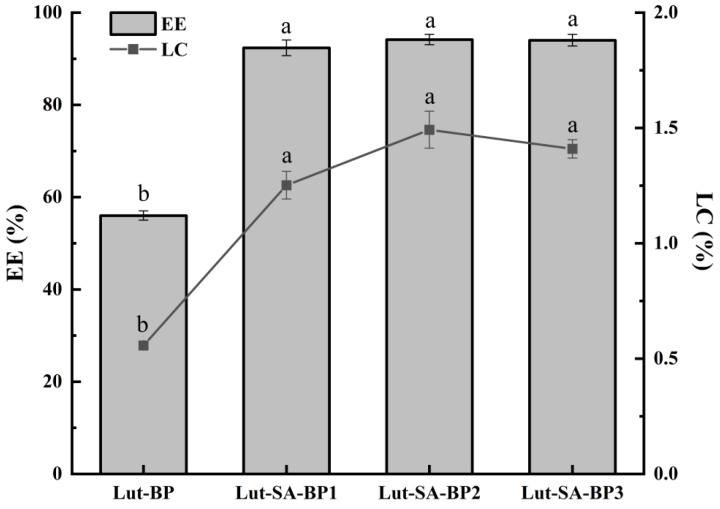
Effect of SA-BP with different DS on the EE and LC of lutein. Different lowercase letters represent significant differences among different groups of the same indicator (*p* < 0.05).

The aggregate carriers prepared with different DS of SA-BP all had high lutein EE and LC (Figure 1). The EE and LC of lutein in Lut-SA-BP2 aggregates were the highest, reaching 94.10% and 1.49%, respectively, which was significantly higher than that of the unmodified BP carrier (*p* < 0.05). This might be attributed to the hydrophobic modification of BPs with stearic acid, which formed amphiphilic polysaccharide aggregates that self-assembled in an aqueous medium to form hydrophobic domains. Hydrophobic lutein was encapsulated in the hydrophobic domains. The hydrogen bonding and hydrophobic interaction between lutein and modified BPs were enhanced. Negahban et al. [24] also observed similar phenomena. The effects of SA-BP with different DS on the EE and LC of lutein were not significant (*p* > 0.05), but if the DS was too high, the hydrophobic domain area of polysaccharide aggregates decreased, and the LC of lutein presented a weakening trend.

### 3.2. Structural Characterization

#### 3.2.1. Microstructure, Particle Size, and Zeta Potential

**Figure 2:**
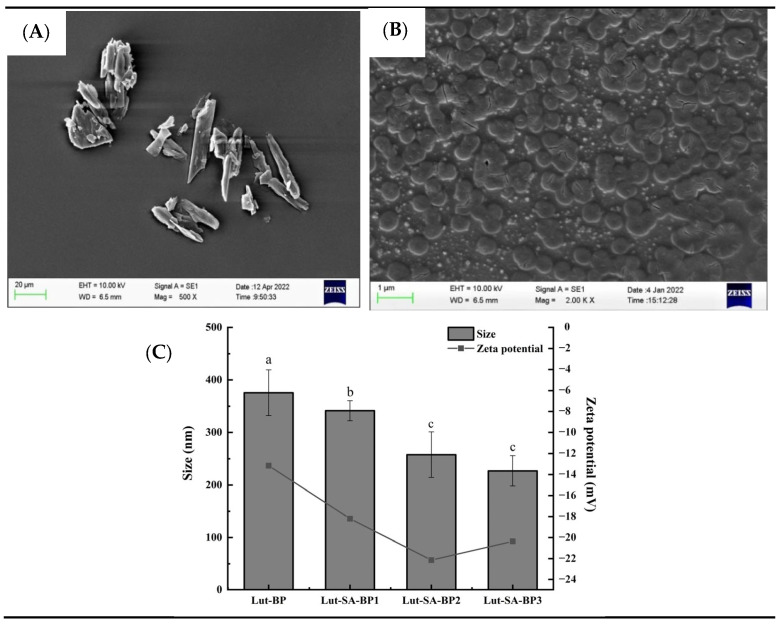
The microstructures of lutein crystals (**A**) and Lut-SA-BP aggregates (**B**), as well as the particle size and zeta potential of Lut-SA-BP aggregates (**C**). Different lowercase letters denote significant differences (*p* < 0.05).

As depicted in Figure 2A,B, lutein crystals were observed by SEM to be in an irregular sheet-like shape, while Lut-SA-BP aggregates appeared approximately spherical; some spheroids gathered, which was consistent with the previous report [25]. It was speculated that lutein entered the hydrophobic domains of SA-BP, yet a small amount of unencapsulated lutein remained. The average particle size and zeta potential of the aggregates loaded with lutein by unmodified BP were 380 nm and −13.4 mV, respectively (Figure 2C). The particle size of the aggregates modified with stearic acid decreased and was negatively correlated with the DS. The absolute value of the potential of the stearic acid-modified aggregates increased, with Lut-SA-BP2 aggregates exceeding 20 mV, indicating that the aggregate surface carried more negative charges, the interparticle repulsive forces were strong, and they exhibited greater stability.

#### 3.2.2. XRD and DSC Analysis

As shown in Figure S2A, the pure lutein characteristic absorption peaks were mainly distributed at 2θ = 12.76°, 13.59°, and 19.99°, exhibiting sharp diffraction peaks in a crystalline state [26]. The physical mixture displayed some weak characteristic peaks of lutein, indicating that the mixture did not change the crystal form. However, lutein-loaded SA-BP aggregates revealed relatively flat amorphous diffuse peaks, suggesting the transition of lutein from a crystalline to an amorphous state, indicating effective encapsulation. A similar result was documented by Li et al. [27] during the encapsulation of lutein in zein-soluble soybean polysaccharide composite nanoparticles.

As observed in Figure S2B, with the increase in DSC temperature, the lutein powder underwent a phase transition at 58.03 °C. An exothermic peak appeared near 103.29 °C, which might be related to the degradation of lutein at high temperatures. A strong endothermic peak appeared near 158.52 °C, representing the melting point of lutein crystals [28]. The glass transition point of BP was located at 114.26 °C, while the modified SA-BP2 showed a higher glass transition temperature of 140.12 °C, indicating improved thermal stability of the modified BP. Additionally, the melting peaks of Lut-BP and Lut-SA-BP2 near 158.52 °C disappeared, suggesting that the specific crystal form of the encapsulated lutein was disrupted and existed in an amorphous form [29], which was consistent with the XRD analysis result.

#### 3.2.3. FTIR and FS Analysis

The Lut-SA-BP2 were characterized by FTIR, as shown in Figure S3A. Lutein corresponded to the stretching vibration absorption peaks of –OH and –OH-related at 3384 and 2960 cm^−1^, respectively; the symmetric –CH_2_ stretching vibration and the asymmetric –CH_3_ stretching vibration absorption peaks at 2914 and 2848 cm^−1^, respectively; and the C=C stretching vibration absorption peak at 1715 cm^−1^, which was a typical absorption peak of lutein crystals in the range of 1900–1650 cm^−1^ [30]. SA-BP2 had a broad absorption peak at 3262 cm^−1^, which was generated by the stretching vibration of –OH; there was a C–H stretching vibration absorption peak at 2914 cm^−1^; the strong absorption peak near 1022 cm^−1^ was caused by the angular vibration of C–O–C or C–O–H [31]. After the embedding of lutein, the absorption peak intensity near 3262 cm-1 of Lut-SA-BP2 became higher, and it was also shifted from 3262 cm^−1^ to 3258 cm^−1^, indicating that a relatively strong hydrogen bond was formed between SA-BP2 and lutein; additionally, some characteristic absorption peaks of lutein disappeared, proving that lutein was encapsulated in the SA-modified BP aggregate [32]. The above results also showed that the structure of SA-BP2 remained largely unchanged after combining with lutein, with hydrophobic interactions between SA-BP2 and lutein primarily involving C–H and O–H bonds. The enhanced hydrophobic interaction between SA-BP and lutein molecules promoted the formation of the aggregate structure.

To further explore the interaction between lutein and different BP derivatives, FS analysis was conducted, as depicted in Figure S3B. Free lutein exhibited a weak fluorescence emission spectrum at 507.5 nm. However, after the formation of aggregates with hydrophobic modified BP, the fluorescence intensity increased, surpassing that of lutein in Lut-BP. Moreover, Lut-SA-BP2 aggregates displayed a more pronounced blue shift effect (from 507.5 nm to 497.6 nm), indicating a stronger hydrogen bond interaction between SA-BP2 and lutein. The heightened hydrophobic groups in SA-BP2 led to an enhanced hydrophobic interaction with lutein.

### 3.3. Storage Stability

**Figure 3:**
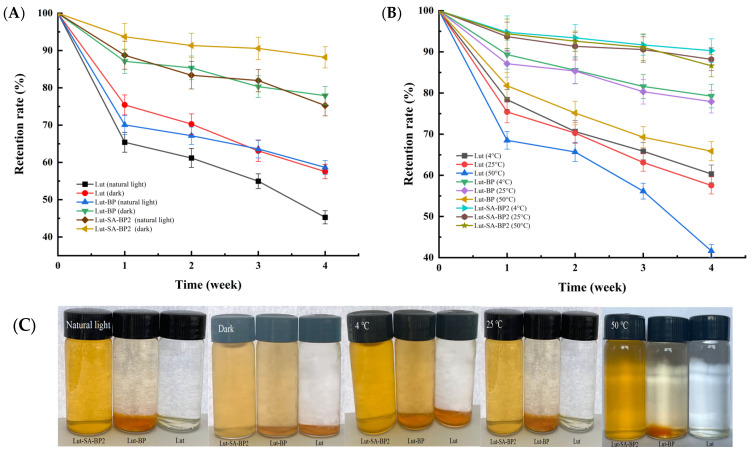
Effects of storage conditions on the retention rate of lutein in Lut-SA-BP2 aggregates. (**A**) The effect of natural light and light avoidance conditions at 25 °C; (**B**) the effect of different storage temperatures (4 °C, 25 °C, and 50 °C) under dark conditions; (**C**) photos of samples corresponding to different storage conditions.

As shown in Figure 3A, lutein crystals degraded rapidly under both natural light and light avoidance conditions, and the retention rate of lutein after four weeks of storage was only 45.27–57.54%. Light exposure may cause the oxidation and cleavage of the C=C bonds in lutein, thereby resulting in epoxidation and promoting hydroxylation reactions, and further leading to precipitation. Whether under light or not, Lut-SA-BP2 aggregate solution remained in a clear and transparent orange-yellow liquid state (Figure 3C), and the light stability of lutein was significantly improved. After four weeks of storage under light and light avoidance conditions, the retention rate of lutein in Lut-SA-BP2 reached 75.27% and 88.19%, respectively (Figure 3A). The hydrophobic domains of the polysaccharide aggregate effectively protected lutein from damage. Shen et al. [33] also found that after 12 h of light treatment, the retention rate of quercetin encapsulated in the amphiphilic chitosan-grafted α-octane sulfonate micelle carrier was 9.33% higher than that of free quercetin.

As shown in Figure 3B, after four weeks of storage at 4 °C, the retention rate of lutein encapsulated by modified BP was increased by 29.26% and 11.04% compared with lutein crystals and Lut-BP. The increase in storage temperature accelerated the Brownian motion between molecules and increased the collision frequency between particles, causing aggregation between particles [34]. However, the Lut-SA-BP aggregates solution remained clear at higher temperatures, with no obvious precipitation at the bottom (Figure 3C). At 50 °C, the retention rates of lutein crystals, Lut-BP, and Lut-SA-BP2 were 43.63%, 65.87%, and 86.62%, respectively, indicating that the retention rate of lutein was significantly improved after encapsulation with modified polysaccharides (*p* < 0.05). This was similar to the result of Ge et al. [35].

### 3.4. In Vitro Release Properties

#### 3.4.1. In Vitro Bioavailability

As shown in Table 1, the bioavailability of lutein in Lut-SA-BP2 aggregates was the highest at 54.72%, significantly surpassing that of Lut-BP (*p* < 0.05) by 3.37 times. This difference could be attributed to the low encapsulation rate of lutein by BP, leading to unembedded lutein dissolving in the upper oil phase or settling at the bottom of the digestive fluid post-digestion. Compared to Lut-SA-BP2 aggregates, the bioavailability of lutein in both Lut-SA-BP1 aggregates and Lut-SA-BP3 aggregates were relatively low, indicating varying impacts of different DS of BP on lutein bioavailability. Notably, the modified BP2 demonstrated superior lutein embedding capabilities, resulting in polymeric aggregates that significantly enhanced lutein bioavailability.

#### 3.4.2. Release Characteristics of Lutein in Simulated Gastrointestinal Fluid

**Figure 4:**
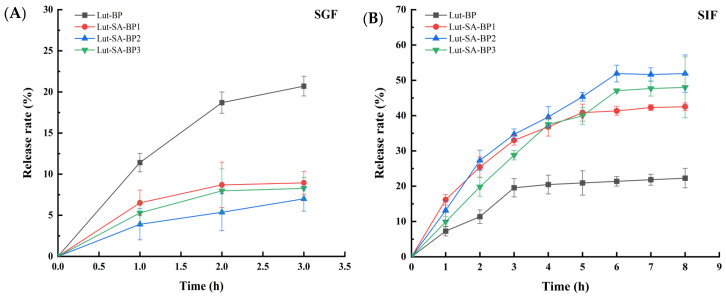
Cumulative release of Lut-SA-BP aggregates in simulated gastrointestinal fluid. (**A**) Simulated gastric juice; (**B**) simulated intestinal fluid.

Figure 4A illustrated that lutein had a rapid initial release within 0–1 h across all four carriers, with Lut-BP showing a notably higher release rate compared to the Lut-SA-BP aggregates. This might be due to the higher concentration of free lutein attached to the BP surface. Between 2 and 3 h, the release of lutein from the three Lut-SA-BP aggregates was slower than that from Lut-BP. After 3 h, the release rate of lutein was less than 10% in all cases. During the slow-release phase, the release of lutein was caused by the dissolution of the aggregate skeleton. The Lut-SA-BP aggregates exhibited a considerably slower release rate than Lut-BP, primarily due to the stronger bond between lutein and the hydrophobic domains of SA-BP, which delayed the release of lutein. As food typically remains in the stomach for 3 to 4 h, an extended simulation of gastric juice digestion tests was not conducted.

As shown in Figure 4B, Lut-BP was released rapidly in the simulated intestinal fluid within the initial 3 h, followed by a gradual increase after 3 h. After 8 h, the release rate of lutein was 22.27%; notably, the cumulative release rates of the three Lut-SA-BP aggregates in the intestinal fluid were significantly higher compared to those in the simulated gastric juice. The cumulative release rate of lutein exhibited a quicker increase within the first 4 h, with a growth rate surpassing that of Lut-BP. This could be attributed to the disruption of the Lut-BP structure in the simulated gastric juice, leading to the early release of lutein and hindering its transition to the intestinal fluid. Additionally, the solubility and dispersibility of the BP carrier were enhanced in the alkaline environment, facilitating the breakdown of the polysaccharide carrier and thereby enhancing the release of lutein. Within 6 to 8 h, the cumulative release rate of lutein gradually decelerated. After 8 h of incubation in the simulated intestinal fluid, the release rate of lutein in Lut-SA-BP2 reached 51.91%. These results indicated that the SA-BP aggregate carrier could exert a controlled release effect on lutein, and improve the stability and intestinal targeting of lutein. Chang et al. [36] also demonstrated that lutein had a slow and sustained release characteristic in the amphiphilic biopolymer aggregate of octenyl succinic anhydride-modified short dextran chains. The above results indicated that the hydrophobic domains inside the amphiphilic polysaccharide aggregate modified with SA effectively protected the structure of the hydrophobic lutein from damage in gastric juice, enabling sustained release.

**Figure 5:**
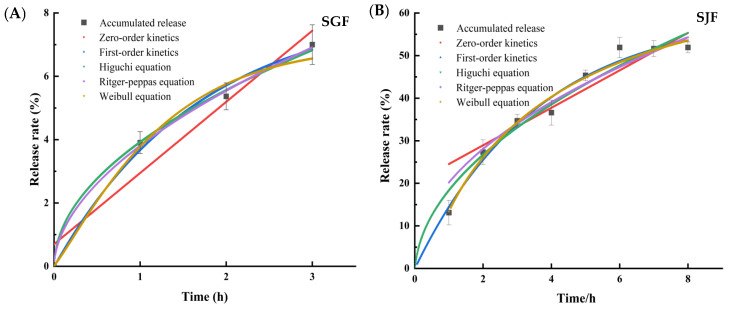
Curve fitting results for the cumulative release of Lut-SA-BP2 aggregate in simulated gastrointestinal fluid. (**A**) Simulated gastric juice; (**B**) simulated intestinal fluid.

The Origin 2021 software was used to analyze the release of lutein in Lut-SA-BP2 in two different release media by fitting the cumulative release amount (Q) with five selected release equations over time (t). Results from Figure 5 and Table 2 indicated that when applying five different kinetic model equations to fit the release of Lut-SA-BP2 in simulated gastric juice, the fitting degree was relatively low except for the zero-order kinetic equation. The first-order kinetic equation, Higuchi planar diffusion equation, Ritger-Peppas equation, and Weibull equation also demonstrated good fitting effects. Particularly, the fitting degree of the Ritger-Peppas equation was the best (R^2^ = 0.9972), with a characteristic parameter b of 0.54, indicating that the release of Lut-SA-BP2 aggregates in simulated gastric juice was influenced by the combination of diffusion and relaxation changes [37]. The release behavior of Lut-SA-BP2 in simulated intestinal fluid was best described by the Weibull model, with a fitting degree of R^2^ = 0.9765. This model was commonly used to explain the release of active compounds from drug delivery systems [38]. According to the Weibull model, lutein release at 8 h exceeded the measured value, suggesting that lutein might continue to be released.

### 3.5. Cellular Osmotic Transport Characteristics

#### 3.5.1. The Cototoxicity

**Figure 6:**
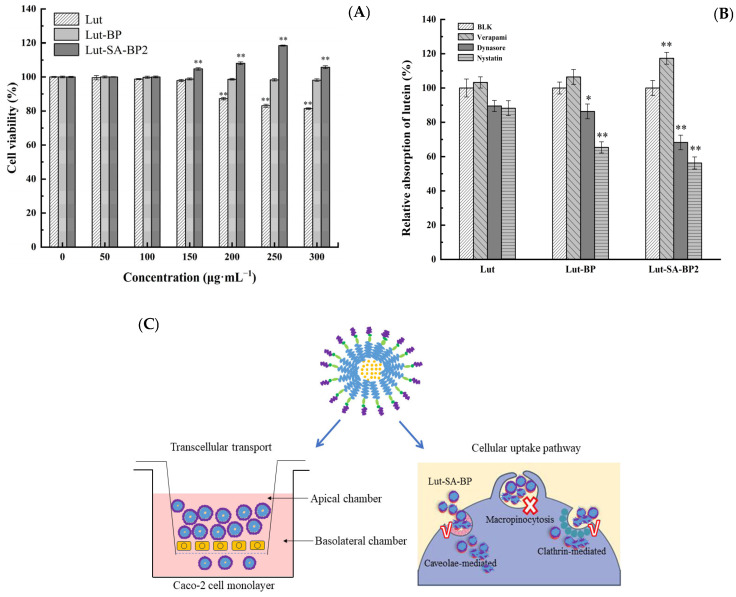
Cell viability (**A**) of Lut-SA-BP2 aggregates, its cellular uptake (**B**) treated with inhibitors and possible uptake pathway (**C**). The asterisk indicates statistical significance (* *p* < 0.05, ** *p* < 0.01).

The viability of Caco-2 cells was determined using the CCK-8 method. As shown in Figure 6A, when three different samples were applied to the Caco-2 cells, it could be seen that the concentration of the sample had a different effect on the cell viability. Generally, when the mass concentration of lutein was 200 µg/mL, the cell viability began to decrease, and when the mass concentration of lutein was 300 µg/mL, the cell viability was 81.36%; the cell viability of Lut-BP within the range of 50–300 µg/mL was all above 98%, and it could be considered that Lut-BP was non-toxic within this concentration range; within the mass concentration range of 50–100 µg/mL of Lut-SA-BP2, the cell viability could reach 100%, when the concentration of Lut-SA-BP2 continued to increase, the cell viability was all above 100%, and when the mass concentration was 250 µg/mL, the cell survival rate was the largest, which was 118.48%. It indicated that the modified amphiphilic polysaccharide was non-toxic to cells and showed a trend of promoting cell growth. The difference in cytotoxicity caused by different forms of lutein might be related to the different ways lutein entered the cell. Free lutein entered the cell by passive transport, while lutein aggregates could accumulate on the cell surface through endocytosis, form new vesicles through plasma membrane invagination, and then enter the cell to achieve cell proliferation [39].

#### 3.5.2. P_app_ and Inhibition of Cellular Uptake

As presented in Table 3, the *P_app_* of lutein, Lut-BP, and Lut-SA-BP2 across the membrane were 1.79 × 10^−5^, 2.20 × 10^−5^, and 5.31 × 10^−5^ cm/s, respectively, among which the *P_app_* of Lut-SA-BP2 was significantly higher than that of the lutein and Lut-BP groups (*p* < 0.05). The *P_app_* values of the three samples were all greater than 1 × 10^−6^ cm/s (reflecting the ease of drug absorption), indicating that the reason affecting the bioavailability of lutein was not the intestinal barrier. The *P_app_* (BL→AP)/*P_app_* (AP→BL) values of lutein, Lut-BP, and Lut-SA-BP2, namely the *P_ratio_* values, were 0.75, 1.20, and 0.45, respectively. The *P_ratio_* value could be used to determine the proportion of drug transport based on passive diffusion or transmembrane transport. Studies have shown that when the *P_ratio_* was greater than 2 or less than 0.5, it indicated the active efflux or uptake mechanism of the drug; when the *P_ratio_* was between 0.5 and 2, it indicated that the transport of the drug in the cell was mainly carried out by passive transport [40]. The *P_ratio_* of lutein and Lut-BP was between 0.5 and 2, which indicated that the transport mode of lutein and Lut-BP was mainly passive transport. The *P_ratio_* of the Lut-SA-BP2 group was below 0.5, thus the transport was an active efflux or uptake mechanism. Compared with lutein, the *P_app_* (AP→BL) value of Lut-SA-BP2 was 2.96 times that of lutein, indicating that the encapsulation of amphiphilic BP aggregates significantly increased the permeability and absorption of lutein in small intestinal epithelial cells.

As shown in Figure 6B, compared with the blank group, the addition of verapamil, which had an inhibitory effect on P-glycoprotein, significantly increased the cellular uptake of Lut-SA-BP2 aggregates (*p* < 0.05), indicating that the transport had a certain competitive effect with the P-glycoprotein inhibitor. Nystatin significantly reduced the cellular uptake of Lut-SA-BP aggregates (*p* < 0.01), reducing by 43.73% compared with the blank group. Dynasore had a significant inhibitory effect on the transport of both SA-BP2 and BP (*p* < 0.05), and the inhibitory effect on SA-BP2 was stronger. Nystatin and dynasore had no inhibitory effect on lutein (*p* > 0.05).

Small molecule substances interacted with the plasma membrane and then entered the cell through different endocytic pathways. Due to the differences in the ways and mechanisms by which different substances entered the cell, endocytosis could be divided into three categories: phagocytosis, pinocytosis, and receptor-mediated endocytosis. Several studies have demonstrated that the absorption of carotenoids in the small intestine is mediated by proteins or passive diffusion mechanisms. On one side of the Caco-2 cell chamber, there was an efflux protein, namely P-glycoprotein, which acted as a transporter and was responsible for transferring the active substances between cells to the outside of the cell, while inhibiting energy consumption and controlling the cell’s absorption efficiency of nutrients.

The addition of verapamil could inhibit the transfer of lutein between cells to the outside of the cell by P-protein, inhibiting the efflux of lutein, thereby increasing the cellular uptake of lutein. Nystatin and dynasore both showed a significant inhibitory effect on the cellular uptake of Lut-SA-BPs aggregates (*p* < 0.05), indicating that the internalization of Lut-SA-BP2 aggregates might be mediated by a combination of the clathrin-dependent endocytic pathway and the caveolae (or lipid raft)-related endocytic pathway (Figure 6C). The internalization of lutein in BP aggregates was mainly achieved through the clathrin-dependent endocytic pathway. Additionally, the uptake of small molecule active substances in intestinal epithelial cells was also related to their particle size. Therefore, defining the exact cellular uptake mechanism of lutein was complex and multiple mechanisms might exist in a single cell. This study provided a basis for further elucidating the cellular uptake mechanism of lutein-loaded polysaccharide-based aggregates.

## 4. Conclusions

A novel BP polymer aggregate was synthesized through an esterification reaction using BP and SA as raw materials. Three different DS of SA-BP were utilized to incorporate lutein, achieving encapsulation efficiencies exceeding 90%. The aggregates were uniformly spherical, with sizes ranging from 227 nm to 341 nm. XRD revealed that lutein existed in an amorphous form in the aggregates. FT-IR and FS analysis demonstrated that lutein entered the hydrophobic domains of SA-BP. The storage stability of Lut-SA-BP2 aggregate solution was notably enhanced. The bioavailability of lutein in Lut-SA-BP2 aggregates was 4.36 times higher than that in unmodified samples. These aggregates exhibited relative stability in gastric fluid and enhanced lutein release in intestinal fluid. By establishing a Caco-2 monolayer cell model, it was shown that the *P_app_* (AP→BL) value of Lut-SA-BPs aggregates was 2.96 times higher than that of lutein alone. The transport of Lut-SA-BP2 aggregates in Caco-2 cells competed with P-glycoprotein inhibitors, primarily facilitating the transmembrane absorption of lutein through caveolae (or lipid raft)-related and clathrin-dependent endocytosis pathways. The above results suggested that SA-BP aggregates hold promise as effective carriers for delivering hydrophobic lutein.

## Figures and Tables

**Table 1:** The bioavailability of Lut-SA-BP aggregates in simulated gastrointestinal fluid.

Sample	Lut-BP	Lut-SA-BP1	Lut-SA-BP2	Lut-SA-BP3
Bioavailability (%)	12.53 ± 3.24 ^c^	36.01 ± 4.32 ^b^	54.72 ± 4.54 ^a^	42.72 ± 3.88 ^b^

Note: Different letters indicate significant differences (*p* < 0.05).

**Table 2:** Fitting results of the kinetic equation for the release curve of Lut-SA-BP2 aggregates.

	Model	Fitted Equation	a	b	R^2^
Simulated gastric juice	Zero-order kinetics	Q(t) = 2.24t + 0.7	2.24	0.70	0.9089
	First-order kinetics	Q(t) = 8.24 (1 − e^−0.59t^)	8.24	0.59	0.9889
	Higuchi equation	Q(t) = 3.96t^0.5^ + 0.04	3.96	0.04	0.9959
	Ritger-Peppas equation	Q(t) = 3.81 (t^0.54^)	3.81	0.54	0.9972
	Weibull equation	Q(t) = 7(1 −$e^{{-\left(0.7958\left(t+0.01\right)\right)}^{1.167}}$)	0.796	1.167	0.9865
Simulated intestinal fluids	Zero-order kinetics	Q(t) = 4.4t + 20.13	4.40	20.13	0.8378
	First-order kinetics	Q(t) = 60.02 (1 − e^−0.18t^)	60.02	0.18	0.9764
	Higuchi equation	Q(t) = 20.25t^0.5^ − 1.97	20.25	1.97	0.9701
	Ritger-Peppas	Q(t) = 18.31 (t^0.55^)	18.31	0.55	0.9504
	Weibull equation	Q(t) = 65.48 (1 − $e^{{-\left(0.267\left(t-0.459\right)\right)}^{0.762}}$)	0.267	0.762	0.9775

**Table 3:** The *P_app_* of Lut-SA-BP2 aggregates.

Sample	*P*_app_/×10^−5^ (cm/s)		$\frac{P_{app}(BL\to AP)}{P_{app}(AP\to BL)}$
	AP→BL	BL→AP	
Lutein	1.79 ± 0.47 ^c^	1.34 ± 0.32 ^c^	0.75 ± 0.03 ^B^
Lut-BP	2.20 ± 0.51 ^b^	2.65 ± 0.24 ^b^	1.20 ± 0.03 ^A^
Lut-SA-BP2	5.31 ± 0.40 ^a^	2.38 ± 0.54 ^b^	0.45 ± 0.02 ^C^

Note: a–c represent the significant differences between different treatment groups in the same direction (*p* < 0.05); A–C represent the significant differences in *P_app_* (BL→AP)/*P_app_* (AP→BL) values between different treatment groups (*p* < 0.05).

## Data Availability

The data supporting this study’s findings are available upon request from the corresponding author.

## Supplementary Materials

The following supporting information can be downloaded at: https://www.mdpi.com/article/10.3390/polym16141982/s1, Figure S1: The synthetic route map of SA-modified BP; Figure S2: XRD (A) and DSC (B) of Lut-SA-BP aggregates; Figure S3: FTIR (A) and FS (B) of Lut-SA-BP2 aggregates.
