# Supplementary material for: Delivery of Lutein by Using Modified Burdock Polysaccharide Aggregates: Preparation, Characterization, and In Vitro Release Properties
Source: Polymers (Basel). 2024 Jul 11;16(14):1982. doi: 10.3390/polym16141982 (PMC11281097; doi:10.3390/polym16141982)
Supplement: Supplementary file 1 [file polymers-16-01982-s001.zip › polymers-3069044-supplementary.pdf]

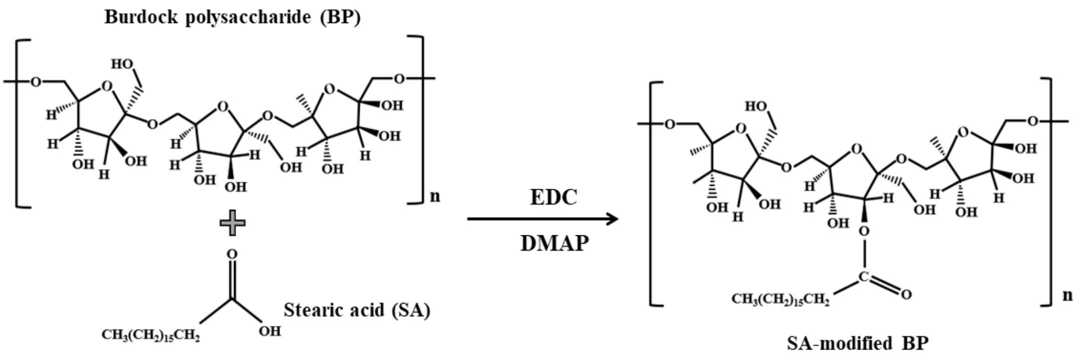

Figure S1 The synthetic route map of SA-modified BP.

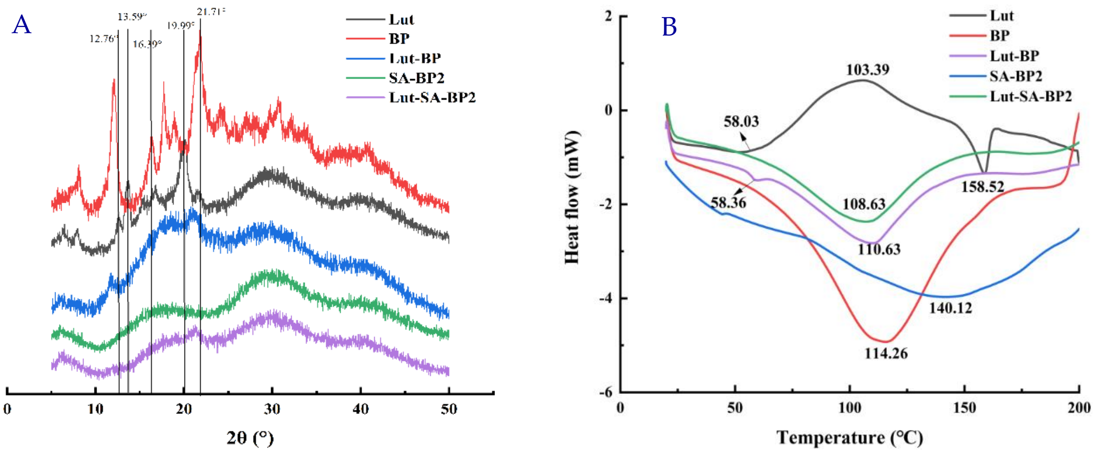

Figure S2. XRD (A) and DSC (B) of Lut-SA-BP aggregates.

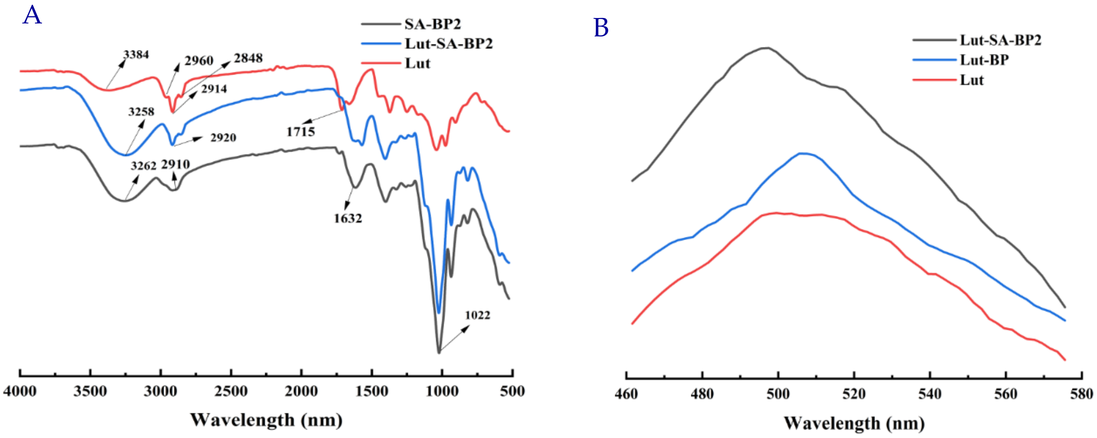

Figure S3. FTIR (A) and FS (B) of Lut-SA-BP2 aggregates.
